# Supplementary material for: Metabolic adaptability in metastatic breast cancer by AKR1B10-dependent balancing of glycolysis and fatty acid oxidation
Source: Nat Commun. 2019 Jun 20;10:2698. doi: 10.1038/s41467-019-10592-4 (PMC6586667; doi:10.1038/s41467-019-10592-4)
Supplement: Supplementary file 1 — Supplementary Information [file 41467_2019_10592_MOESM1_ESM.pdf]

**Metabolic adaptability in metastatic breast cancer by**

**AKR1B10-dependent balancing of glycolysis and fatty acid oxidation**

**Supplemental Material**


| | |
|---|---|
| Supplementary Table 1. | Details of the 23 shortlisted shRNAs significantly depleted in the *in vivo* screen |
| Supplementary Table 2. | Mission shRNA lentiviral transduction particles (Sigma) |
| Supplementary Table 3. | Plasmids for viral production |
| Supplementary Table 4. | Antibodies |
| Supplementary Figure 1. | Downregulation of *Akr1b8* expression has no impact on cell viability *in vitro* |
| Supplementary Figure 2 | Representative lung images from spontaneous metastasis assay |
| Supplementary Figure 3. | *AKR1B10* expression in human breast cancer cell lines and clinical samples from untreated breast cancer patients |
| Supplementary Figure 4. | Lipid peroxidation monitored by FACS |

**Supplementary Table 1. Details of the 23 shortlisted shRNAs significantly depleted in the *in vivo* screen**

**Supplementary Table S1**

| shRNA | Pool | Gene Symbol | Z score (lung sample versus preinoculation cells) | | | | | | Viability | Significant replicates |
|---|---|---|---|---|---|---|---|---|---|---|
| | | | Sample A | Sample B | Sample C | Sample D | Median (A-D) | corrected | | |
| V2MM_1137 | 28 | Bcl2l2 | -4.542 | -5.372 | -4.5252 | -5.8515 | -4.9572 | -4.8046 | 0.7250 | 4 |
| V2MM_48375 | 40 | Bag1 | -1.657 | -5.689 | -4.4224 | -3.6798 | -4.0511 | -3.9614 | 0.3385 | 3 |
| V2MM_74286 | 5 | Rnd3 | -3.402 | -3.782 | -2.2041 | -3.8586 | -3.5921 | -3.6541 | -0.5945 | 4 |
| **V2MM_73415** | **35** | **Akr1b8** | **-3.555** | **-1.513** | **-4.0938** | **-3.6325** | **-3.5938** | **-3.5493** | **0.0601** | **3** |
| V2HS_112625 | 1 | Btf3 | -4.863 | -5.258 | -2.0982 | -1.6365 | -3.4804 | -3.5478 | -0.6281 | 3 |
| V2MM_31175 | 24 | Orc3l | -3.983 | -4.375 | -2.7538 | -3.3050 | -3.6440 | -3.4830 | 0.7766 | 4 |
| V2HS_28393 | 11 | Stk17b | 0.001 | -3.719 | -3.7463 | -3.9061 | -3.7329 | -3.4787 | 1.3496 | 3 |
| V2MM_52437 | 30 | Mmp9 | -4.566 | -0.028 | -4.1067 | -2.0374 | -3.0721 | -3.0686 | -0.1924 | 3 |
| V2MM_70338 | 16 | Ctsd | -2.857 | -3.193 | -2.6871 | -2.2471 | -2.7719 | -2.9473 | -1.2922 | 4 |
| V2MM_72419 | 40 | Rarg | -1.398 | -3.261 | -3.3903 | -2.8587 | -3.0598 | -2.9097 | 0.7096 | 3 |
| V2MM_63946 | 14 | Igf1 | -3.620 | -4.768 | -2.2023 | -1.2261 | -2.9110 | -2.7948 | 0.5008 | 3 |
| V2MM_36205 | 8 | Rfc4 | -0.722 | -3.066 | -2.5579 | -2.4643 | -2.5111 | -2.6971 | -1.3577 | 3 |
| V2MM_78883 | 16 | Fosl2 | -3.320 | -2.145 | -2.8100 | -0.9707 | -2.4772 | -2.6823 | -1.4745 | 3 |
| V2MM_52206 | 32 | Ccnc | -2.977 | -2.856 | -0.6731 | -2.5510 | -2.7037 | -2.5749 | 0.5789 | 3 |
| V2MM_30812 | 32 | Met | -2.787 | -3.813 | -1.7972 | -2.5040 | -2.6454 | -2.5513 | 0.3652 | 3 |
| V2MM_215947 | 42 | Sox15 | -1.001 | -2.470 | -3.0020 | -2.8376 | -2.6539 | -2.5265 | 0.5699 | 3 |
| V2MM_49133 | 29 | Mapk8 | -2.723 | 0.067 | -2.5966 | -2.3775 | -2.4870 | -2.4924 | -0.2466 | 3 |
| V2MM_70791 | 14 | Nol3 | 0.849 | -2.515 | -2.3605 | -4.0625 | -2.4377 | -2.4847 | -0.5027 | 3 |
| V2MM_46386 | 22 | Fzd1 | -2.182 | -2.798 | -2.9950 | -1.6952 | -2.4899 | -2.4548 | 0.0027 | 3 |
| V2MM_9444 | 16 | Bard1 | -2.128 | -2.514 | -1.1018 | -2.8216 | -2.3212 | -2.2868 | -0.0017 | 3 |
| V2MM_64342 | 12 | Rhob | -2.072 | -0.604 | -2.3696 | -4.1559 | -2.2206 | -2.2734 | -0.5381 | 3 |
| V2MM_51741 | 31 | Prkch | -2.296 | 2.216 | -3.0748 | -2.1568 | -2.2265 | -2.1264 | 0.4021 | 3 |
| V2MM_82583 | 43 | Cast | -2.327 | 0.581 | -2.4447 | -2.1373 | -2.2321 | -2.0470 | 0.9248 | 3 |

Pool = library subpool; Significant replicates =  number of technical replicates with Z score <-2; Viabilty = preinoculation cells versus plasmid library

**Supplementary Table 2. Mission shRNA lentiviral transduction particles (Sigma)**

| Clone ID | Gene target | NM ID | Name |
|---|---|---|---|
| SHC001V | N/A | N/A | shCTRL |
| SHC216V | None | N/A | shNTC |
| TRCN0000042204 | Akr1b8 | NM_008012 | shAkr1b8-4 |
| TRCN0000042207 | Akr1b8 | NM_008012 | shAkr1b8-7 |

**Supplementary Table 3. Plasmids for viral production**

| Plasmid ID | Vector | Supplier | Description | Selection marker |
|---|---|---|---|---|
| psPAX2 | psPAX2 | | Mammalian expression, lentiviral packaging | N/A |
| pMD2.G | pMD2.G | | Mammalian expression, lentiviral envelope | N/A |
| RHS4346 | pGIPZ | Dharmacon | non-targeting shRNA (shNTC) | Puromycin |
| V3LHS-400714 | pGIPZ | Dharmacon | AKR1B10 shRNA | Puromycin |
| EX-NEG-Lv156 | pReceiver-Lv156 | Genecopoeia | Vec (empty vector) | Puromycin |
| EX-S0319-Lv156 | pReceiver-Lv156 | Genecopoeia | AKR1B10 open reading frame | Puromycin |

## Supplementary Table 4. Antibodies

| Antibody | Species | Source (catalogue number) | Assays | Dilution |
|---|---|---|---|---|
| Anti-α-tubulin | Mouse | Sigma Aldrich (B-5-1-2) | WB | 1:1000 |
| Anti-AKR1B10 | Mouse | Abcam (ab57547) | WB | 1:500 |
| Anti-human lamin A/C | Rabbit | Abcam (ab108595) | IHC | 1:500 |
| IRDye 680RD Donkey anti-Mouse IgG | Donkey | Li-Cor (926-68072) | WB | 1:10000 |
| IRDye 800CW Donkey anti-Mouse IgG | Donkey | Li-Cor (926-32212) | WB | 1:10000 |
| IRDye 800CW Donkey anti-Rabbit IgG | Donkey | Li-Cor (926-32212) | WB | 1:10000 |

IHC, immunohistochemistry; WB, western blot

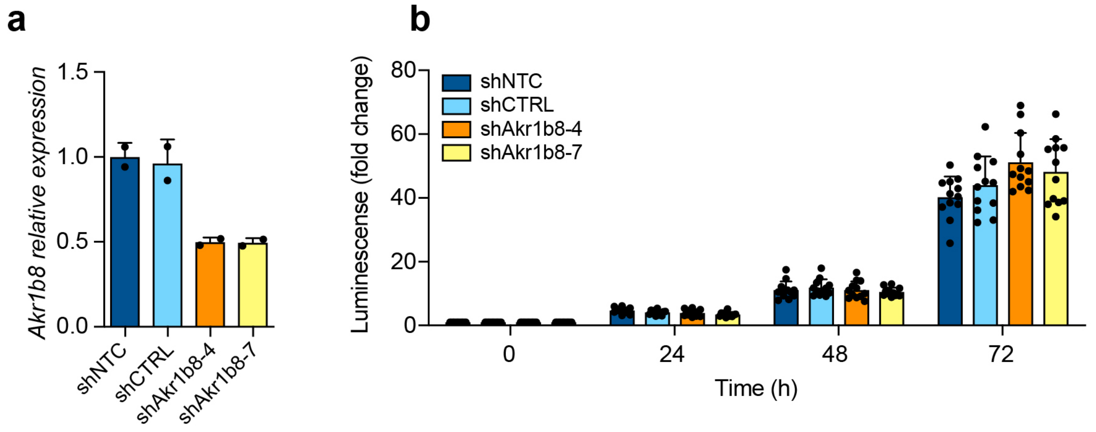


**Supplementary Figure 1.** Downregulation of *Akr1b8* expression has no impact on cell viability *in vitro*

4T1-Luc cells were transduced with empty pLKO.1-puro vector particles (shCTRL), a non-targeting shRNA control (shNTC) or two independent shRNA lentiviral transduction particles targeting shAkr1b8-4 and shAkr1b8-7. **a**, Real-time RT-qPCR analysis of *Akr1b8* expression (TaqMan FAM probe Mm0437762_m1) normalised to *B2m* expression (TaqMan FAM probe Mm00484314_m1). Data shown are mean RQ relative expression levels from 2 independent experiments (3 technical replicates each) ±SD. **b,** 2x$10^2$ cells from each cell line were plated per well of a 96-well plate. Every 24 hours, beginning 1 hour post plating, viability was quantified by CellTiter-Glo assay and plotted as fold change as compared to the initial reading. n=12 wells per sample per time point. Data shown are the mean fold change compared to the 1 hour time point.

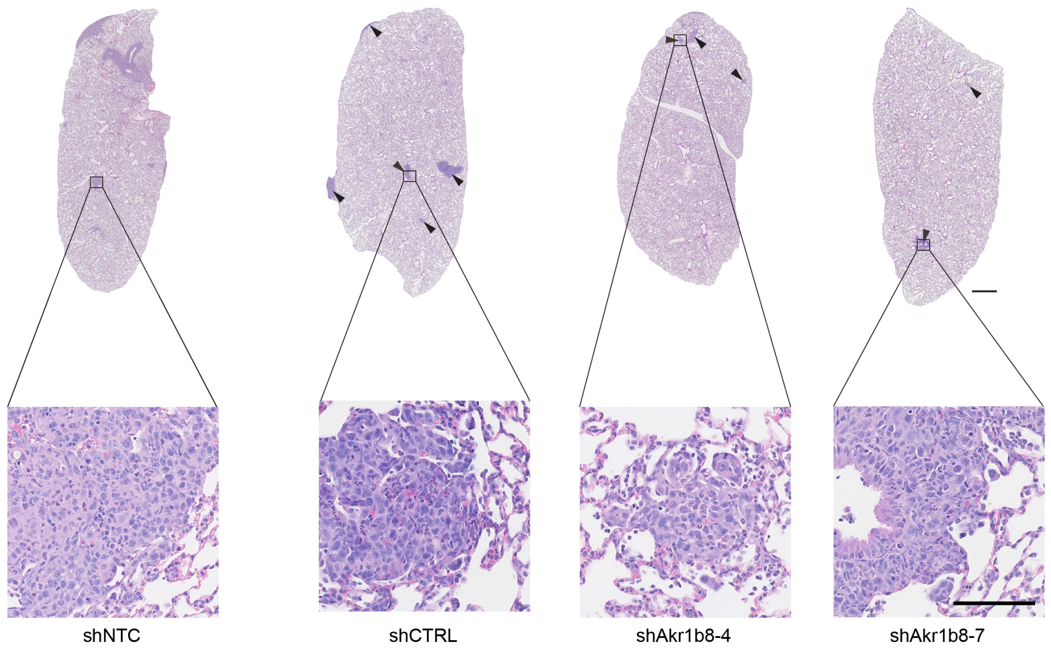


**Supplementary Figure 2.** Representative lung sections from spontaneous metastasis assay shown in Figure 1e.

Representative lung sections from 4T1 spontaneous metastasis assay in BALB/c mice (Fig. 1e). Arrowheads indicate tumour nodules. Lower power images, scale bar = 1 mm. Higher power images, scale bar = 100 µm.

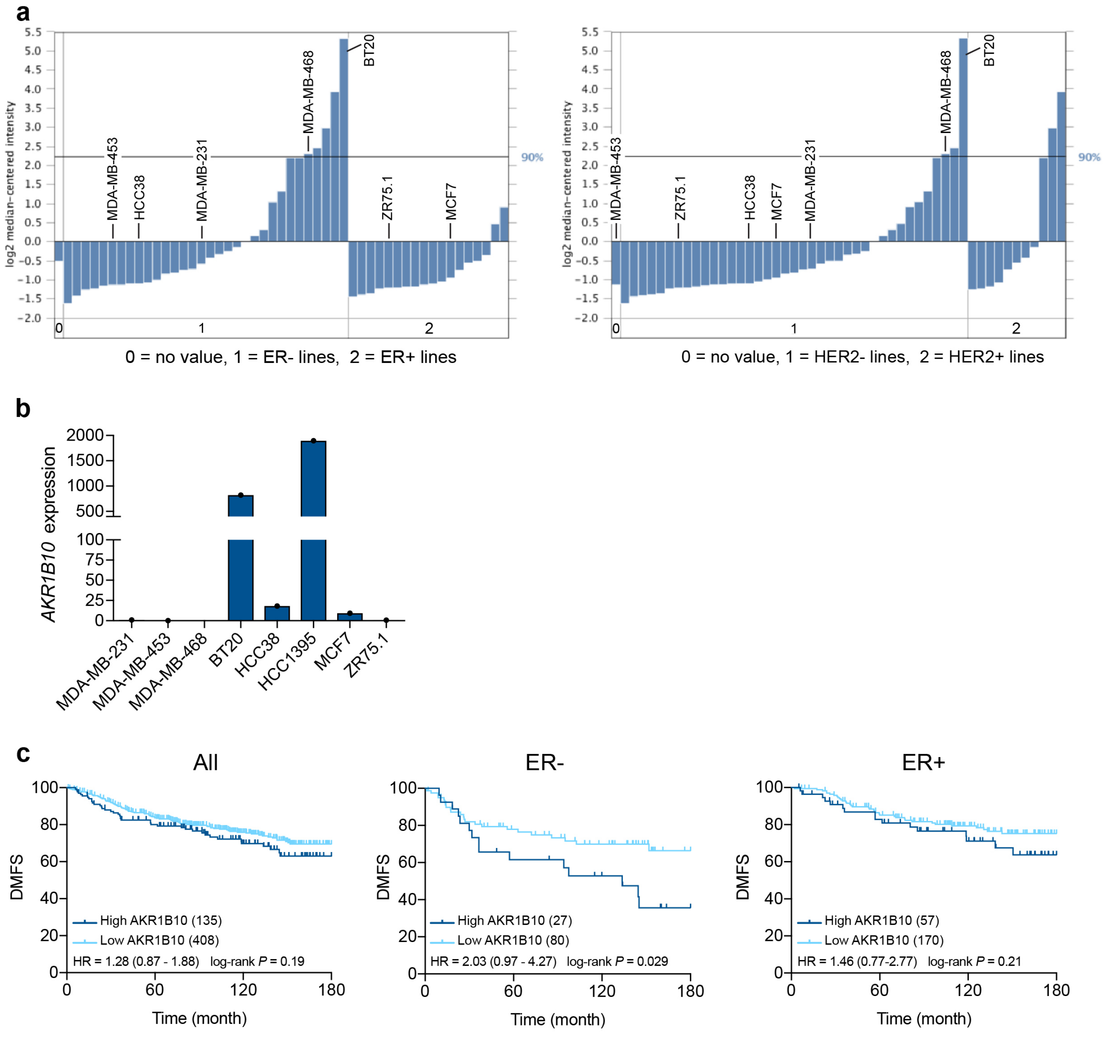


**Supplementary Figure 3.** *AKR1B10* expression in human breast cancer cell lines and clinical samples from untreated breast cancer patients.

**a,** Expression of *AKR1B10* in the Neve *et al.* breast cancer cell line dataset separated by ER and HER2 receptor status. Cell lines used in Fig. 2b are indicated. **b**, RT-qPCR analysis of *AKR1B10* expression (TaqMan FAM probe Hs00252524_m1) normalised to *GAPDH* expression (TaqMan VIC probe 4352932E) in the cell lines used in Fig. 2b. **c,** Kaplan-Meier analysis of distant metastasis-free survival (DMFS) of the 538 untreated breast cancers in the Gyorffy *et al*., dataset, or in the subsets of ER- untreated breast cancers (n=107) or ER+ untreated breast cancers (n=227). Hazard ratios (HR) and log-rank Mantel-Cox *P*-values are shown.

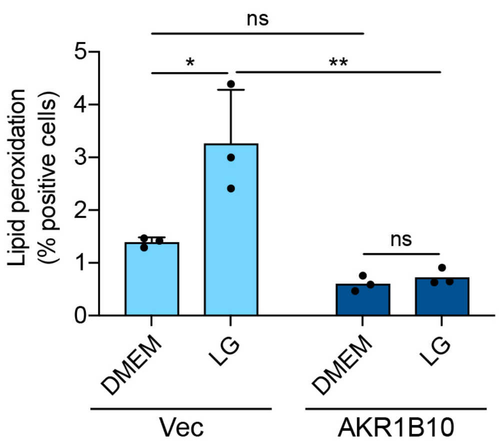


**Supplementary Figure 4.** Lipid peroxidation monitored by FACS

1x10$^5$ MDA-MB-231 cells transduced with vector-alone (Vec) or ectopically expressing AKR1B10 were cultured for 48 h in regular DMEM or low glucose (LG) DMEM plus 10% FBS before staining with BODIPY 581/591 C11 Image-iT (C10445; Molecular Probes) at 37°C for 30 min. Cells were washed, DAPI staining and FACS analysed. Data show % cells with oxidised (green) lipid probe. n=3 samples per condition ±SD.
