# Supplementary material for: Metabolic adaptability in metastatic breast cancer by AKR1B10-dependent balancing of glycolysis and fatty acid oxidation
Source: Nat Commun. 2019 Jun 20;10:2698. doi: 10.1038/s41467-019-10592-4 (PMC6586667; doi:10.1038/s41467-019-10592-4)
Supplement: Supplementary file 2 — Reporting Summary [file 41467_2019_10592_MOESM2_ESM.pdf]

nature research

Corresponding author(s): Clare M Isacke

Last updated by author(s): May 2, 2019

Reporting Summary

Nature Research wishes to improve the reproducibility of the work that we publish. This form provides structure for consistency and transparency in reporting. For further information on Nature Research policies, see Authors & Referees and the Editorial Policy Checklist.

Statistics

For all statistical analyses, confirm that the following items are present in the figure legend, table legend, main text, or Methods section.

n/a	Confirmed	
☐	☒	The exact sample size (*n*) for each experimental group/condition, given as a discrete number and unit of measurement
☒	☐	A statement on whether measurements were taken from distinct samples or whether the same sample was measured repeatedly
☐	☒	The statistical test(s) used AND whether they are one- or two-sided<br>*Only common tests should be described solely by name; describe more complex techniques in the Methods section.*
☒	☐	A description of all covariates tested
☐	☒	A description of any assumptions or corrections, such as tests of normality and adjustment for multiple comparisons
☐	☒	A full description of the statistical parameters including central tendency (e.g. means) or other basic estimates (e.g. regression coefficient) AND variation (e.g. standard deviation) or associated estimates of uncertainty (e.g. confidence intervals)
☐	☒	For null hypothesis testing, the test statistic (e.g. *F*, *t*, *r*) with confidence intervals, effect sizes, degrees of freedom and *P* value noted<br>*Give P values as exact values whenever suitable.*
☒	☐	For Bayesian analysis, information on the choice of priors and Markov chain Monte Carlo settings
☒	☐	For hierarchical and complex designs, identification of the appropriate level for tests and full reporting of outcomes
☐	☒	Estimates of effect sizes (e.g. Cohen's *d*, Pearson's *r*), indicating how they were calculated

Our web collection on statistics for biologists contains articles on many of the points above.

Software and code

Policy information about availability of computer code

Data collection	Not Applicable
Data analysis	*Provide a description of all commercial, open source and custom code used to analyse the data in this study, specifying the version used OR state that no software was used.*

For manuscripts utilizing custom algorithms or software that are central to the research but not yet described in published literature, software must be made available to editors/reviewers. We strongly encourage code deposition in a community repository (e.g. GitHub). See the Nature Research guidelines for submitting code & software for further information.

Data

Policy information about availability of data

All manuscripts must include a data availability statement. This statement should provide the following information, where applicable:

- Accession codes, unique identifiers, or web links for publicly available datasets
- A list of figures that have associated raw data
- A description of any restrictions on data availability

Details of the datasets analysed in this study are included in the section of “Analysis of human datasets”. The source data (uncropped immunoblots) underlying Fig. 2b are provided as Source Data File 1.xlsx.

Field-specific reporting

Please select the one below that is the best fit for your research. If you are not sure, read the appropriate sections before making your selection.

☒ Life sciences ☐ Behavioural & social sciences ☐ Ecological, evolutionary & environmental sciences

For a reference copy of the document with all sections, see nature.com/documents/nr-reporting-summary-flat.pdf

Life sciences study design

All studies must disclose on these points even when the disclosure is negative.

Sample size	Sample sizes for in vivo experiments were based on laboratory experience; for example more mice were used in spontaneous metastasis assays as they are more variable than experimental metastasis assays.
Data exclusions	No data were excluded
Replication	For in vivo experiments, equivalent data was obtained in two independent experiments or by dye-swap experiments. For in vitro assays, the majority of experiments were repeated on more than one occasion and this has been stated per experiment in the figure legends. In other cases, data were validated by including multiple cell lines
Randomization	In all animal experiments mice were randomized based on individual mouse body weights at the start of the experiment
Blinding	not applicable

Reporting for specific materials, systems and methods

We require information from authors about some types of materials, experimental systems and methods used in many studies. Here, indicate whether each material, system or method listed is relevant to your study. If you are not sure if a list item applies to your research, read the appropriate section before selecting a response.

Materials & experimental systems

n/a	Involved in the study	
☐	☒	Antibodies
☐	☒	Eukaryotic cell lines
☒	☐	Palaeontology
☐	☒	Animals and other organisms
☒	☐	Human research participants
☒	☐	Clinical data

Methods

n/a	Involved in the study	
☒	☐	ChIP-seq
☒	☐	Flow cytometry
☒	☐	MRI-based neuroimaging

Antibodies

Antibodies used	Information on antibodies used are provided in Supplementary Table 4.
Validation	AKR1B10 antibody was validated in the shRNA knockdown cells as shown in Fig 2b.<br>Human specific lamin A/C antibody was validated in Fig 2e, this antibody only reacts with human cells and not with normal mouse lung tissue.

Eukaryotic cell lines

Policy information about cell lines

Cell line source(s)	4T1 cells were obtained from ATCC in 2013 and transduced with firefly luciferase lentiviral expression particles to generate 4T1-Luc cells. HEK293 cells and human breast cancer cell lines (MDA-MB-231, MDA-MB-231-Luc, MDA-MB-453, MDA-MB-468, BT20, HCC38, HCC1395) were obtained from ATCC.
Authentication	Cell lines tested using short tandem repeat every 4 months (StemElite ID System; Promega).
Mycoplasma contamination	Cell lines were routinely subject to mycoplasma testing.
Commonly misidentified lines<br>(See ICLAC register)	not applicable

Animals and other organisms

Policy information about studies involving animals; ARRIVE guidelines recommended for reporting animal research

Laboratory animals	6-8 week old female BALB/c or BALB/c Nude (CAnN-Cg-Foxn1nu/Crl) mice used in this study and were purchased from Charles River.
Wild animals	not applicable
Field-collected samples	not applicable
Ethics oversight	All animal work was carried out under UK Home Office Project licenses 70/7413 and P6AB1448A (Establishment License, X702B0E74 70/2902) and was approved by the Animal Welfare and Ethical Review Body at The Institute of Cancer Research.

Note that full information on the approval of the study protocol must also be provided in the manuscript.
